# Pancreatic stone protein as a biomarker for the early diagnosis of post-operative peritonitis, intra-abdominal infection and sepsis

**DOI:** 10.1093/jscr/rjac497

**Published:** 2022-11-14

**Authors:** François Ventura, Yvan Gasche, Aymen Kraiem Ben Rached, Déborah Pugin, Frédéric Mollard, Samir Vora, Pierre Charbonnet, Léo Bühler

**Affiliations:** Division of Critical Care Medicine, Hirslanden Clinique des Grangettes, Geneva, Switzerland; Division of Anesthesiology, University Hospitals of Geneva, Geneva, Switzerland; Division of Critical Care Medicine, Hirslanden Clinique des Grangettes, Geneva, Switzerland; Division of Critical Care Medicine, Hirslanden Clinique des Grangettes, Geneva, Switzerland; Division of Critical Care Medicine, Hirslanden Clinique des Grangettes, Geneva, Switzerland; Division of Critical Care Medicine, Hirslanden Clinique des Grangettes, Geneva, Switzerland; Division of Infectiology, Hirslanden Clinique des Grangettes, Geneva, Switzerland; Division of Surgery, Hirslanden Clinique des Grangettes, Geneva, Switzerland; Division of Surgery, Hirslanden Clinique des Grangettes, Geneva, Switzerland; Surgical Research Unit, University of Fribourg, Fribourg, Switzerland

## Abstract

The diagnosis of intra-abdominal infection and post-operative peritonitis based on clinical examination, biomarkers and radiological signs, should be made as early as possible to improve outcomes and decrease mortality through early and optimal source control, adequate surgery and appropriate antibiotic therapy (Montravers *et al*. Therapeutic management of peritonitis: a comprehensive guide for intensivists. *Intensive Care Med* 2016;**42**:1234–47). However, the indication and the timing of the surgery is often not an easy decision. This case presents the use of a novel early biomarker of infection and sepsis, pancreatic stone protein (Fidalgo *et al*. Pancreatic stone protein: review of a new biomarker in sepsis. *J Clin Med* 2022;**11**:1085), as a tool to aid in the diagnosis of intra-abdominal infection and post-operative peritonitis and to help guide the decision for adequate surgeries in a patient with intra-abdominal infection and post radical prostatectomy peritonitis.

## INTRODUCTION

Complications of abdominal surgery with intra-abdominal infection, post-operative peritonitis, sepsis, septic shock and multiple organ failure require complex management and multiple surgical interventions [1,2]. Despite improvement of therapeutic approaches, mortality remains high at 30% in intensive care settings [3].

This case report illustrates the use of serial pancreatic stone protein (PSP) measurements, a new biomarker of infection and sepsis, aiding the diagnosis of recurrent intra-abdominal infection and post-operative peritonitis, and as a warning signal aiding the indication for multiple repeat operations during a long stay in the intensive care unit (ICU).

## CASE REPORT

A 62-year-old patient with no previous medical history was admitted to the hospital for elective radical prostatectomy by robotic laparoscopy. The surgery was performed without any immediate complications.

**Figure 1:**
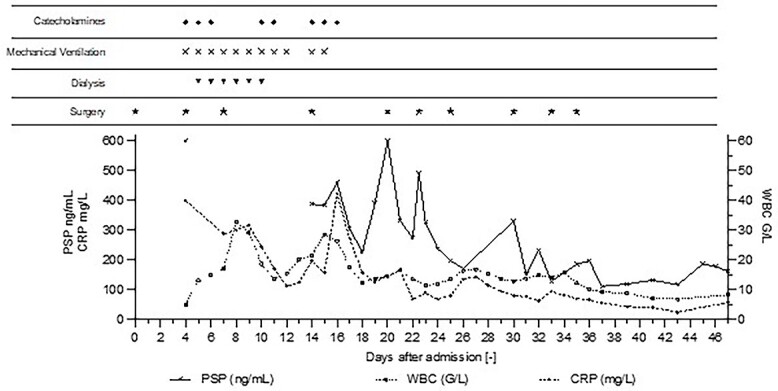
Post-operative Day 1—The patient underwent elective prostatectomy by laparoscopy assisted by robot. Day 4—ICU-admission with severe septic shock and multiple organ failure (PSP > 600 ng/ml). Emergency surgery show intra-abdominal infection and diffuse purulent post-operative peritonitis. Mechanical ventilation, RRT, and catecholamine support were implemented. Day 14—High PSP >387 ng/ml meant the patient was returned to surgery, which revealed loosening of the ileal anastomosis and recurrent diffuse post-operative peritonitis, despite no clinical signs or symptoms, and without clear radiological sign on abdominal CT-scan (Fig. 2). Day 20—High PSP levels (> 600 ng/ml) prompted a new abdominal CT-scan and immediate surgery, confirming suspicion of an intra-abdominal infection, despite stable levels of CRP and WBC. Day 22—PSP measured in the morning and again in the afternoon showed a rapid and twofold increase leading to a new surgery (intraperitoneal lavage) for intra-abdominal infection. Day 30—PSP level 330 ng/ml corresponds to a new intra-abdominal infection despite daily lavage. Day 47—Patient’s condition improved, biomarker levels decreased to stable values, patient was discharged from the ICU.

On post-operative Day 4, the patient presented with severe abdominal pain, high levels of inflammation and infection biomarkers (C-reactive protein CRP > 400 mg/l, PSP > 600 ng/ml; Fig. 1). An abdominal computed tomography (CT) scan showed free intraperitoneal fluid. The patient underwent emergency laparoscopy and then midline laparotomy for diffuse purulent post-operative peritonitis and two ileal perforations were diagnosed (at 3 and 4 m from the Treitz angle). After lavage with 20 l of saline, a distal ileostomy was initially performed on the first perforation (at 3 m of the Treitz) and an anastomotic resection on the second perforation. Following the surgery, the patient was transferred intubated to the ICU, in severe septic shock and multiorgan failure with acute kidney injury. Antibiotic therapy with imipenem/cilastine was started and *Staphylococcus aureus* and *Streptococcus anginosus* were detected in the peritoneal fluid cultures 48 h later.

**Figure 2:**
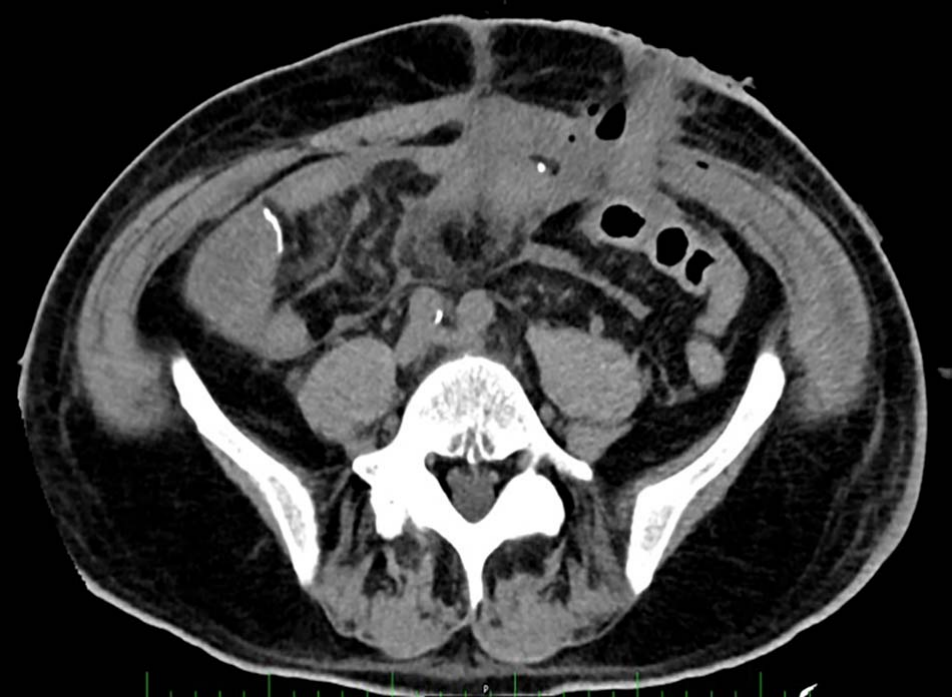
Post-operative Day 14. Abdominal CT-scan with free intraperitoneal fluid, without sign of ileus, perforation or anastomosis release.

On Day 7, a planned exploratory surgery was performed with intraperitoneal lavage and drainage, and a wall vacuum assisted closure VAC implemented. Antimicrobial therapy with Fluconazole was empirically added for 7 days.

Renal replacement therapy was required until Day 10, mechanical ventilation and catecholamines support until Day 12. On Days 13 and 14, the patient did not present any fever or abdominal pain, CRP levels were at 125 mg/l and 196 mg/l, respectively and white blood cell (WBC) counts were stable at 20 G/L. Blood levels of PSP were determined to be 387 ng/ml on Day 14, measured on the point-of-care abioSCOPE® device (Abionic SA, Epalinges, Vaud, Switzerland). This high concentration of PSP (>290 ng/ml [4]) triggered an abdominal CT-scan which showed free fluid, no sign of ileus, perforation or anastomosis release (Fig. 2). Despite this result and absence of clinical signs, the patient was rescheduled for surgery based on the high PSP value. The laparotomy revealed a loosening of the ileal anastomosis and confirmed the presence of a diffuse post-operative peritonitis. A second distal ileostomy was performed along with abundant peritoneal lavage, abdominal wall VAC. *Escherichia coli, Enterococcus faecalis* and *S. aureus* were detected in the peritoneal fluid cultures 48 h later, and antibiotic therapy with imipenem/cilastine/fluconazole was maintained. From Day 14 onwards, PSP, CRP and WBC were dosed daily. These three biomarkers progressively decreased to reach values of 225 ng/ml, 157 mg/l and 12.3 G/L, respectively on Day 18.

**Figure 3:**
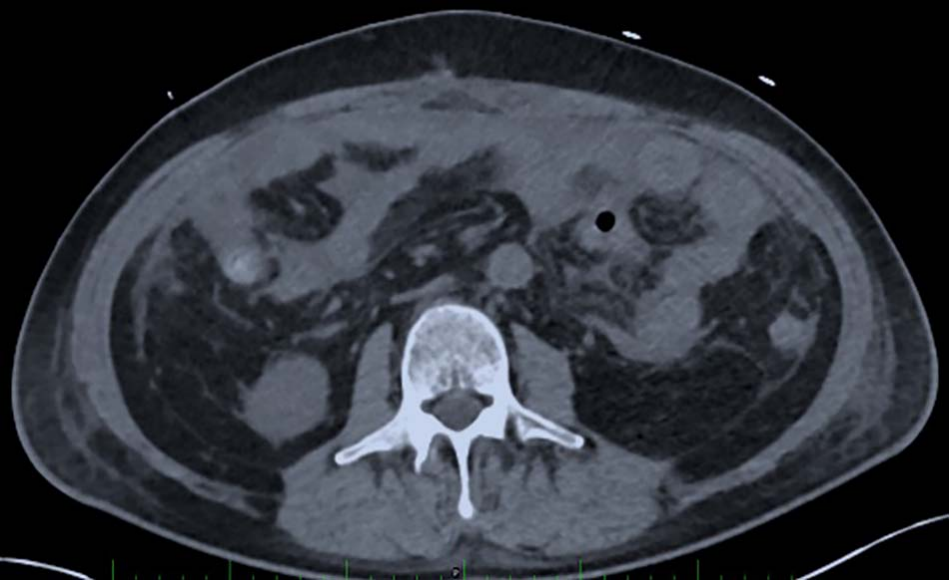
Post-operative Day 20. Abdominal CT-scan with free intraperitoneal fluid, without sign of ileus, perforation or anastomosis release.

On Days 19 and 20, the patient reported no pain, with only transient febrile episodes at 38.1°C during the night. Abdominal examination was normal, and stomas, wall VAC and dressings (which were repeated daily under sedation) did not show any issues. CRP and WBC counts were stable. In contrast to the apparent clinically stable picture, PSP dosing showed elevated concentrations of 392 ng/ml and >600 ng/ml on Days 19 and 20 respectively. Based on this, a new abdominal CT-scan was performed and showed several intraperitoneal fluid collections of radiological density suggestive of an intra-abdominal infection (Fig. 3). The patient was returned to the operating room and surgery confirmed intra-abdominal infection and post-operative peritonitis with purulent collections (cultures will show *E. coli* 48 h later). An intraperitoneal lavage was performed, the abdomen was kept open and a laparostomy using VAC with continuous intraperitoneal lavage was put into place.

On Days 21 and 22, PSP, CRP and WBC counts decreased. In the afternoon of Day 22, the patient reported chills and presented fever at 38.1°C, the PSP level had nearly doubled compared to the morning of the same day (from 273 to 492 ng/ml). The patient immediately underwent a new intraperitoneal lavage for intra-abdominal infection but without a prior abdominal CT-scan.

After Day 22, the patient’s condition improved with daily abdominal lavage in the ICU under sedation. In the absence of identified germs, the abdominal wall was closed on post-operative Day 26. The high PSP level 330 ng/ml on Day 30 (CRP 79 mg/l, WBC 12.9 G/L) corresponds to an abdominal wall infection (*E. coli, Stenotrophomonas maltophilia, Proteus vulgaris, Klebsiella pneumoniae* and *E. faecalis*) despite the daily wall lavage. From Day 30 onwards, the patient’s evolution was favorable with regular abdominal wall lavages and the patient was discharged from the ICU after 47 days.

## DISCUSSION

The cornerstone for effective treatment of intra-abdominal infection and peritonitis requires early recognition and adequate source control, and today the indication for surgery is based on numerous clinical, biological and radiological parameters [5].

Biomarkers such as CRP, white blood cell (WBC) counts, procalcitonin (PCT) and interleukins have been studied as biomarkers to aid the diagnosis of intra-abdominal infections and sepsis. However, their values for identifying abdominal sepsis have yielded conflicting results [6]. A recent literature review [7] of 23 studies including a prospective multi-center study [4] and a meta-analysis [8] suggests that PSP has a higher diagnostic performance for the identification of infection and sepsis than the most used available biomarkers. The multi-center study shows that serial routine PSP measurement has the potential of ‘pre-symptomatic diagnosis of sepsis’ up to 72 h in advance with an optimal PSP cut-off of 290 ng/ml [4]. A prospective cohort study on patients admitted to ICU concluded that serum-PSP levels demonstrate a high diagnostic accuracy to discriminate the severity of peritonitis and to predict mortality in the ICU [9].

## CONCLUSION

This case report illustrates the ability of the novel biomarker PSP concentrations to act as a warning sign, allowing the early identification of intra-abdominal infection and post-operative peritonitis episodes, even prior to their clinical diagnosis (pre-symptomatic diagnosis of sepsis). PSP facilitated prompt surgery in this case report. Further studies can help to fully understand the role of PSP in intra-abdominal infections and post-operative peritonitis.

## CONFLICT OF INTEREST STATEMENT

The corresponding author is Chief Medical Officer at Abionic SA.

## FUNDING

None.
