# Correction to: Pancreatic stone protein as a biomarker for the early diagnosis of post-operative peritonitis, intra-abdominal infection and sepsis

**DOI:** 10.1093/jscr/rjac633

**Published:** 2022-12-28

This is a correction to: François Ventura, Yvan Gasche, Aymen Kraiem Ben Rached, Déborah Pugin, Frédéric Mollard, Samir Vora, Pierre Charbonnet, Léo Bühler, Pancreatic stone protein as a biomarker for the early diagnosis of post-operative peritonitis, intra-abdominal infection and sepsis, *Journal of Surgical Case Reports*, Volume 2022, Issue 11, November 2022, rjac497, https://doi.org/10.1093/jscr/rjac497

In the originally published version of this manuscript, the conflict of interests statement was incorrect. The following should have been declared: the corresponding author is Chief Medical Officer at Abionic SA.

This error has been corrected online.

